# Additively manufactured microwave sensor for glucose level detection in saliva

**DOI:** 10.1038/s41598-024-79867-1

**Published:** 2024-11-15

**Authors:** Ilona Piekarz, Kacper Skarzynski, Blanka Piekarz, Krzysztof Wincza, Slawomir Gruszczynski, Marcin Sloma, Jakub Sorocki

**Affiliations:** 1 Institute of Electronics, AGH University of Krakow, Krakow, Poland; 2 Institute of Metrology and Biomedical Engineering, Warsaw University of Technology, Warsaw, Poland; 3 Department of Applied Computer Science and Modelling, AGH University of Krakow, Krakow, Poland

**Keywords:** 3D printing, Additive manufacturing, Biosensor, Coplanar waveguide, Interdigital capacitor, Metal-on-glass, Microwave sensors, Glucose detection in saliva, Electrical and electronic engineering, Biomedical engineering, Mechanical engineering

## Abstract

In this paper, a novel realization of an ink-on-glass microwave sensor for biomedical applications is proposed. The Aerosol Jet Printing (AJP) technology is leveraged to implement a compact single-layer coplanar waveguide sensor featuring arc-shaped interdigital fingers that can accommodate a droplet of the Material-Under-Test (MUT). Such geometry provides a high sensitivity to even a very small deviation of MUT`s electrical properties when placed as a superstrate. An application towards the detection of trace amounts of glucose in saliva, which is a biomarker for diabetes, is showcased. The design and fabrication process of an exemplary sensor is discussed in detail. A circular geometry feature is introduced that helps a droplet to lie over the sensitive region due to wettability difference of glass substrate and silver ink. Sensor operating in K-band is developed providing a tradeoff between circuit size and droplet volume. The study is conducted for an artificial saliva requiring roughly a 0.5 µL droplet where changes in mixture content are proportional to relative changes of sensor`s transmission coefficient in a broad frequency range for occupied vs. empty states. The obtained results show that 10 mg of glucose per 100 ml of saliva can be easily distinguished in a frequency range of 20–30 GHz, whereas a monotonical change is visible for frequencies 20–26 GHz, which indicates the applicability of this sensor towards the detection of saliva-glucose levels and potential application in the detection of small amounts of other substances in liquids.

## Introduction

Chronic diseases such as diabetes, heart disease, cancer, etc. are the leading cause of death in the United States^1^. Diabetes Mellitus, which is a chronic metabolic disease, occurs when the pancreas does not produce enough insulin or when the body cannot effectively use the insulin it produces. The symptoms of diabetes may occur suddenly and if not early diagnosed and then appropriately controlled can damage blood vessels in the heart, eyes, kidneys, and nerves. Among all the chronic diseases diabetes is the eighth leading cause of death^2^, whereas in the United States, over 37 million people have diabetes^3^. The primary control strategies include the monitoring of blood glucose levels. Unfortunately, traditional blood glucose monitoring methods usually require blood sampling operations. Blood extraction is painful and presents the risk of infection and mental pain to patients, especially for those patients who need to check their blood glucose levels several times a day^4^-^5^ and is especially inconvenient for children and older people. Thus, the investigation of methods allowing for non-invasive blood glucose level monitoring is an important issue^6–11^ and is highly demanded.

Generally, the non-invasive methods are based on the measurement of glucose concentration from its chemical, electrical, optical, and other properties^9^. Among methods utilizing the electrical properties of glucose for its concentration detection, the optical and microwave ones are of great interest. Both methods rely on the quantification of glucose molecules, that exhibit distinct characteristics in the frequency range from a few MHz to THz. By analyzing the interaction of electromagnetic or optical signals with tissues or fluids one can determine the biosample composition, including glucose concentration. The non-invasive or minimally-invasive techniques can be divided into two categories i.e., techniques monitoring blood glucose through skin layers^9,10,12–16^ and methods utilizing other biological materials for glucose level monitoring, which can be collected with minimal discomfort such as tears^17–19^, saliva^5,11,20–22^, exhaled air^7,23^, sweat^24^, etc. In all mentioned methods, the results need to be correlated with direct blood glucose measurements. Tears can be used for continuous monitoring of glucose levels by integration of sensing devices in contact lenses^17–19^. When coupled with electrochemical analysis, such sensors can provide continuous and quantitative glucose monitoring. Nevertheless, the correlation between tear glucose and blood glucose level is controversial due to the variation in the composition of tears depending on the tear collection method^17,25–27^, since stimulated tears can feature different tear compositions than non-stimulated ones. The techniques shown in^9,10,15,16^ have on one hand a great advantage which is the possibility of continuous blood glucose monitoring, however, their sensitivity and reliability are limited by the signal-to-noise ratio level and skin thickness. Among methods allowing for glucose monitoring based on bodily fluids, the ones utilizing saliva for diagnostic purposes have received much attention, since saliva is considered an ultrafiltrate of blood^5^. Moreover, the great advantage of such methods is that the sample can be collected by anyone since saliva is the most accessible biofluid and easy to collect^28^ as well as the risk of sample contamination is low^11^. There is a good correlation between blood glucose and salivary glucose, which was revealed by many studies^6,22,29^. Taking all the above into account, salivary glucose can be utilized as an alternative diagnostic tool for diabetes. Nevertheless, the sensing element must feature high sensitivity because glucose concentration in saliva of a diabetic person is several tens of times lower than in the blood^30,31^. According to^16,30^ normal glucose levels in saliva should be less than 2 mg per 100 ml.

In this paper, we present a novel realization of an ink-on-glass microwave sensor for biomedical applications. The demonstrator circuit was additively manufactured on a glass substrate using the aerosol jet printing technology, which features less material wastage than conventional manufacturing processes^32^. The sensor operates in K-band as a trade-off between fabrication resolution and minimal sized leading to sensitive part occupying only 1.23 × 0.98 mm. In such a way, the sensor has a potential to be easily integrated with semiconductor-on-glass active readout electronics, making it well suited for cost-effective lab-on-chip solution. The geometry of the sensor was chosen to maximize sensitivity on the droplet MUT covering the sensing area, through an in-series arched-wire interdigital capacitor geometry that confines most of the electric field in the measured sample. Moreover, due to difference between substrate and metal wettability, the droplet al.igns itself over the sensing part and is held by a metal ring thus minimizing spill of liquid outside where a potential passive and active electronics might be placed. The manufactured sensors were utilized for the detection of glucose levels in synthetic saliva. The obtained results prove a high sensitivity of the sensor for the detection of glucose being as low as 10 mg per 100 ml of saliva, thus proving its usefulness for future utilization in saliva glucose detection systems.

## Microwave sensor realization

### Sensor design

**Fig. 1:**
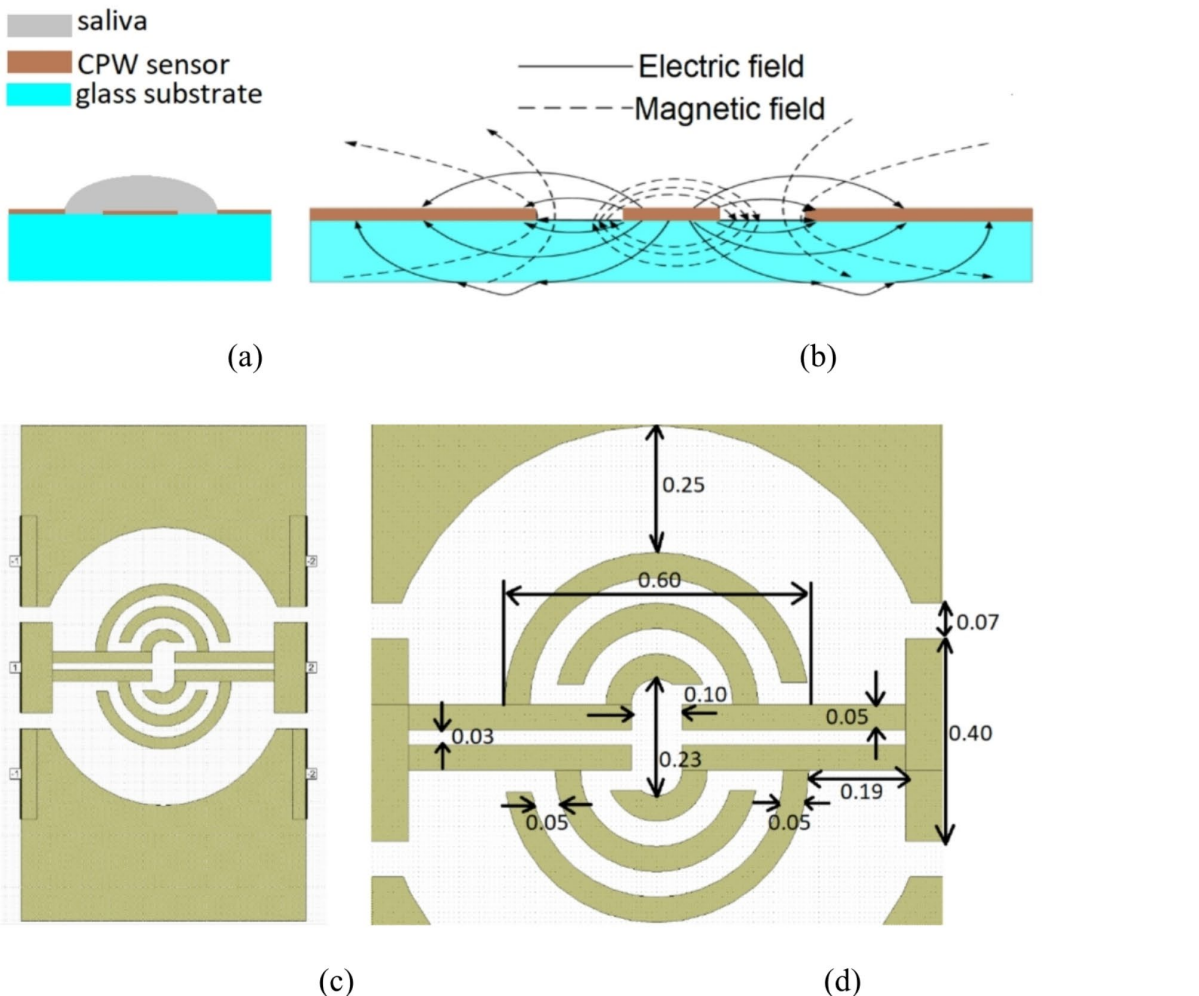
General view on the sensor designed using CPW technique and covered with liquid sample (**a**) and electric field lines showing the greatest current density in the structure (**b**). Top-view on the sensing element (**c**) with a close-up view on the layout with marked most important dimensions in millimeter (**d**).

The sensor is foreseen to be used for the detection of glucose below 2 mg per 100 ml, thus special care needs to be taken to develop a geometry featuring highest possible frequency response change to small changes of complex permittivity of saliva-glucose mixtures. A single-layer coplanar (CPW) waveguide technique was chosen for the realization of the sensor, since such a construction allows to pour the measured liquid material directly on the sensing element (see Fig. 1a), enables low-cost realization as well as great possibility for integration with other electronics. Moreover, the largest amount of the electric field is concentrated between the co-planarly located center conductor and ground planes (see Fig. 1b), thus most of the electric field is enclosed within the sample covering the sensor. Saliva is composed of around 99% water; thus, it features similar properties to water and when stretched (i.e., provided using a laboratory pipette) a drop starts to formulate^33^. Therefore, the following requirements were stated regarding the sensor’s geometry:

- a sensing element need to have a circular shape to accommodate a droplet outline,
- a sensing element need to provide high sensitivity on significantly small changes on the complex permittivity of saliva-glucose mixtures,
- a MUT droplet needs to lie over the sensing region in a reproducible manner.

Taking all of this into account, sensor layout shown in Fig. 1c was developed. The sensor was designed on a 0.26 mm thick glass substrate featuring a relative permittivity of *ε*_*r*_ = 6. The frequency of operation was chosen to be K-band, since higher frequency of operation allows to obtain smaller circuit dimensions and possibility of integration with semiconductor-on-glass active readout electronics in lab-on-chip solutions. Moreover, with the increase of sensor’s operation frequency the changes of complex permittivity of saliva-glucose mixtures should be more visible. There are two reasons behind it i.e., first the permittivity of water being the main saliva component decreases at higher frequencies^34^; second with the increase of glucose amount the complex permittivity of water-glucose solution tends to increase, since more polar molecules are available to respond to the electric field and it is more visible with the increase of frequency^35^. To promote and contain a droplet in a desired spot, radial ground planes were located 625 μm apart from the central sensing part. The droplet formation feature relies on the fact that glass is relatively hydrophilic^36^ and thus wetted by the sample while silver layer is relatively hydrophobic^37^ and so it forces the sample to take the desired shape over the sensing area (when sample volume is small enough not to overcome the surface tension). Moreover, the geometrical features within the silver barrier have rather small area as compared to glass surface to further suit both mechanical and electrical objectives. Moreover, the metal surface is rather rough (in the range of µm) further magnifying the desired effect. A close-up view of the final design with marked major dimensions is presented in Fig. 1d. The sensing part occupies 1.23 ×  0.98 mm.

**Fig. 2:**
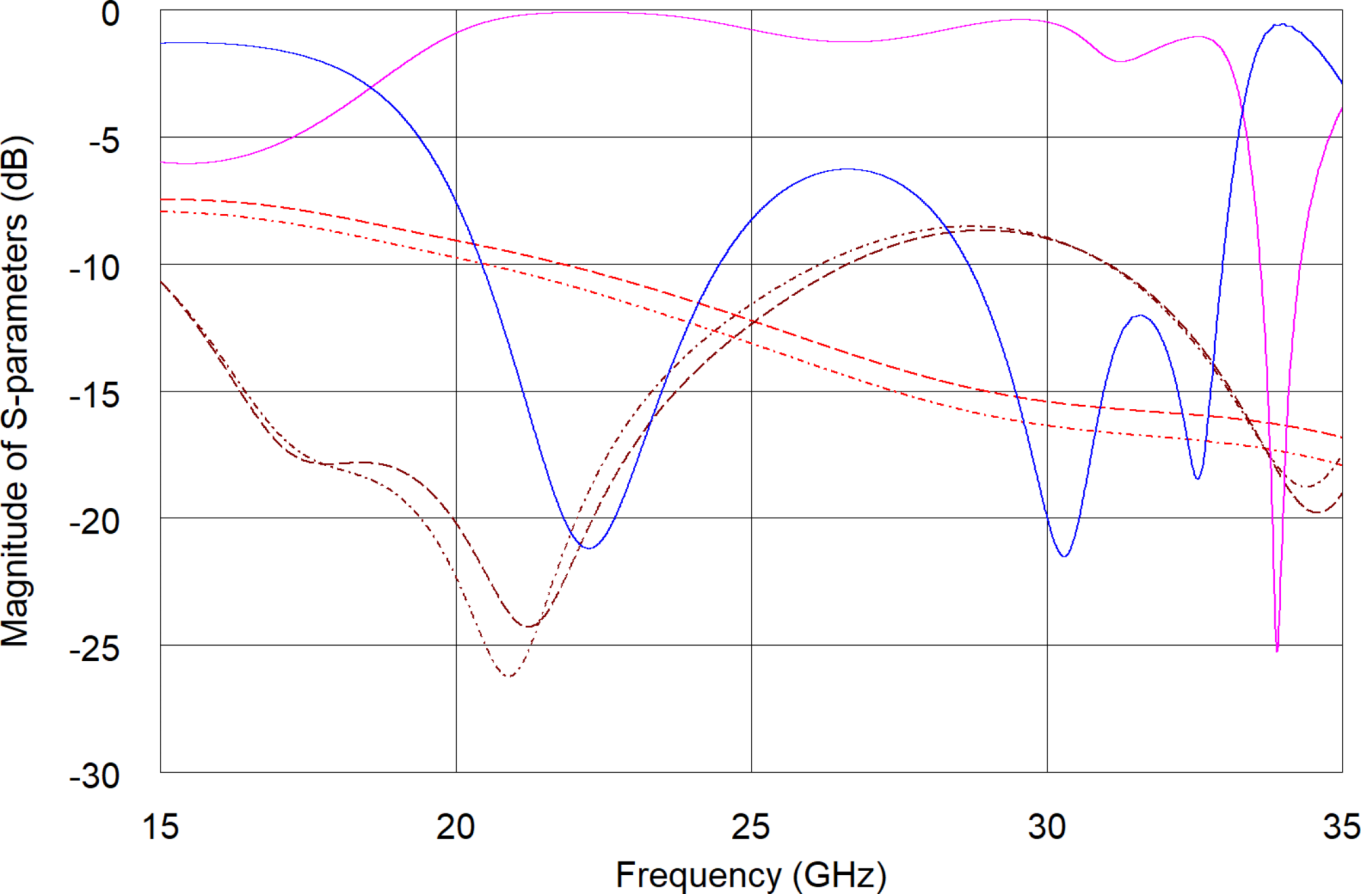
Comparison of EM simulated magnitude of S-parameters of the sensor with no sample on top (solid lines), covered with 0.49 mm thick layer of water (dashed lines) and 2 mg per 100 ml water-glucose solution (dotted lines). Blue and brown lines—reflection, pink and red lines—transmission.

The sensor was electromagnetically (EM) analyzed using A*WR Design Environment* software and the obtained S-parameters are shown in Fig. 2 (solid lines) in a broad frequency range for a sensor with no cover (assuming perfect conductor, lossless substrate) and added transmission lines simulating the measurement probes. As seen, the sensor`s response features multiple resonances in proximity to each other. This is due the sensor shape being multiple arced inter coupled fingers of decreasing lengths towards sensors central symmetry axis, leading to an equivalent circuit being a multiple RLC tanks with increasing resonant frequency (shorter finger length – reduced series capacitance). However, only the fundamental resonance frequency region is to be used for further data analysis as it features the least transmission attenuation and most of the EM field is confined with the outer most semi-circle fingers.

Finaly, the EM model was used to assess quantitively the potential impact of sample presence in the sensing region. Since the goal is to detect 2 mg per 100 ml glucose in saliva, the complex permittivity value of such a solution was estimated based on^34,35^ and taken for the EM simulation. For that purpose, the complex permittivity data was taken from^34^ where 80 mg per 100 ml water-glucose solution was characterized. Knowing that saliva has comparable properties as water due to its composition, water vs. water-glucose impact approximation is valid for this purpose. Permittivity of a 2 mg per 100 ml water-glucose solution was estimated through interpolation assuming a linear relation to be 42.8–17.3j at 25 GHz. On the other hand, complex permittivity of water at 25 GHz is 39.5–34.6j^35^, both at room temperature. The comparison of the sensor frequency response obtained for water (dashed lines) and a water-glucose mixture (dotted lines) is provided in Fig. 2 for a MUT having thickness of 0.49 mm (half of the shorter sensing area diameter). As seen, the transmission characteristics are significantly attenuated in comparison to the characteristics obtained for an empty sensor since water is a highly lossy material. Nevertheless it is seen based on the frequency response that such small concentrations of glucose could be differentiated.

### Sensor manufacturing through aerosol jet printing

For the experiments a set of developed sensor units was fabricated using an aerosol jet printer with an ultrasonic atomizer. The AJP technology is an additive manufacturing process utilizing atomized inks, allowing for selective material deposition. Due to this fact, AJP is an environmentally friendly manufacturing technique that reduces material waste. The important advantage of this technique is the maskless, digital printing approach, which is ideal for small batch production, rapid prototyping, and fast modification of the sample topology in experiments. Conductive straight lines can be printed using the AJP technique featuring a width as low as 6 μm^38^, but the typical width of lines featuring conductivity comparable to bulk silver using conventional AJP setup is 20 μm. The conductivity of aerosol jet printed printouts is in the same order of magnitude as bulk silver^39^ and using experimental reactive inks it can reach the conductivity of plated copper^40^. The cross-section of highly conductive printed lines has a triangular shape. Broad-printed patterns require the distance between the printed lines to be small enough to blend the lines. However, the top surface of the resulting pattern is only partially flat, which results in visible toolpaths and affects the shape of the edge of the printouts. Ultrasonic atomization approach and 150 μm nozzle were used to achieve the high resolution printed patterns. UT Dots’ Ag25Te silver ink exhibiting the properties suitable for high frequency applications^41^ was used to prepare conductive patterns on the glass substrates. The carrier and sheath gas flows, 11.5 sccm and 100 sccm, were selected experimentally to ensure a constant quality of printed patterns in long printing sessions. To control the position of the Aerosol Jet Printing head and glass substrate, the XYZ table Asymtek Dispensemate D593 was used. Sensor design requires a 50 μm line width and arcs with a 50 μm radius. Such a geometry requires significantly lower printing speeds of 0.2 mm/s than typically used 1–8 mm/s printing speed to achieve a 20 μm width line. Higher speed values result in a distortion of the arc geometry, and lower speed values result in poor repeatability or arc length due to the limitations of the positioning mechanisms of the printer. Some minor imperfections in the printed pattern are observed: a narrow line in 1/4 of the arc length and a slight overflow of ink at the very end - these two do not affect significantly the final quality and parameters of the printed pattern. Low printing speed also influences the width of printed lines, allowing wider lines, up to 50 μm, in a single pass. Eventually, the final pattern consisted of two straight lines and an arc of 100 μm radius connecting them. The four-layer samples of 50 μm width were selected as the most optimal for further experiments.

**Fig. 3:**
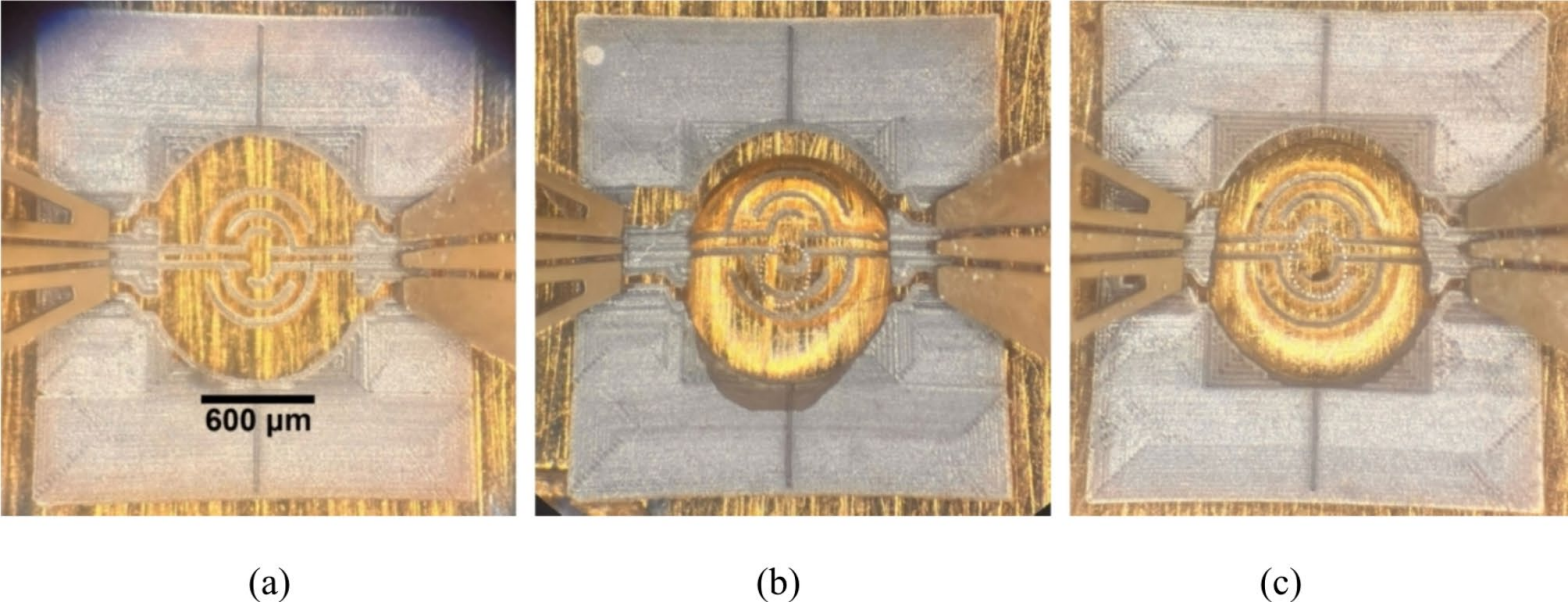
Photographs of manufactured sensor units: empty (**a**) and after dosing a drop of saliva-glucose solution (**b**,**c**).

In the process of samples fabrication, the glass substrates were prepared prior printing by submerging in the acid bath, deionized water, base bath, and deionized water, followed by 5-min cleaning in Ulsonix proclean 2.0 M ultrasonic cleaner. The substrate was then dried with a dust-free paper towel and an air pistol. The substrate was mounted on an aerosol jet printer table by shape-tailored clamping. After deposition, green samples were sintered using a 500 W halogen lamp for 1 min. No extra surface treatment steps were taken past the necessary ones for printing. Sensors need to withstand basic handling to be usable. Therefore, the quality of the samples was verified by gently rubbing them with the cotton cloth and top-view optical inspection using a Motic D-EL2 digital microscope. Photographs of exemplary manufactured units are shown in Fig. 3. As seen, the printed sensing element features consistent lines with relatively sharp edges.

### Experimental study

**Fig. 4:**
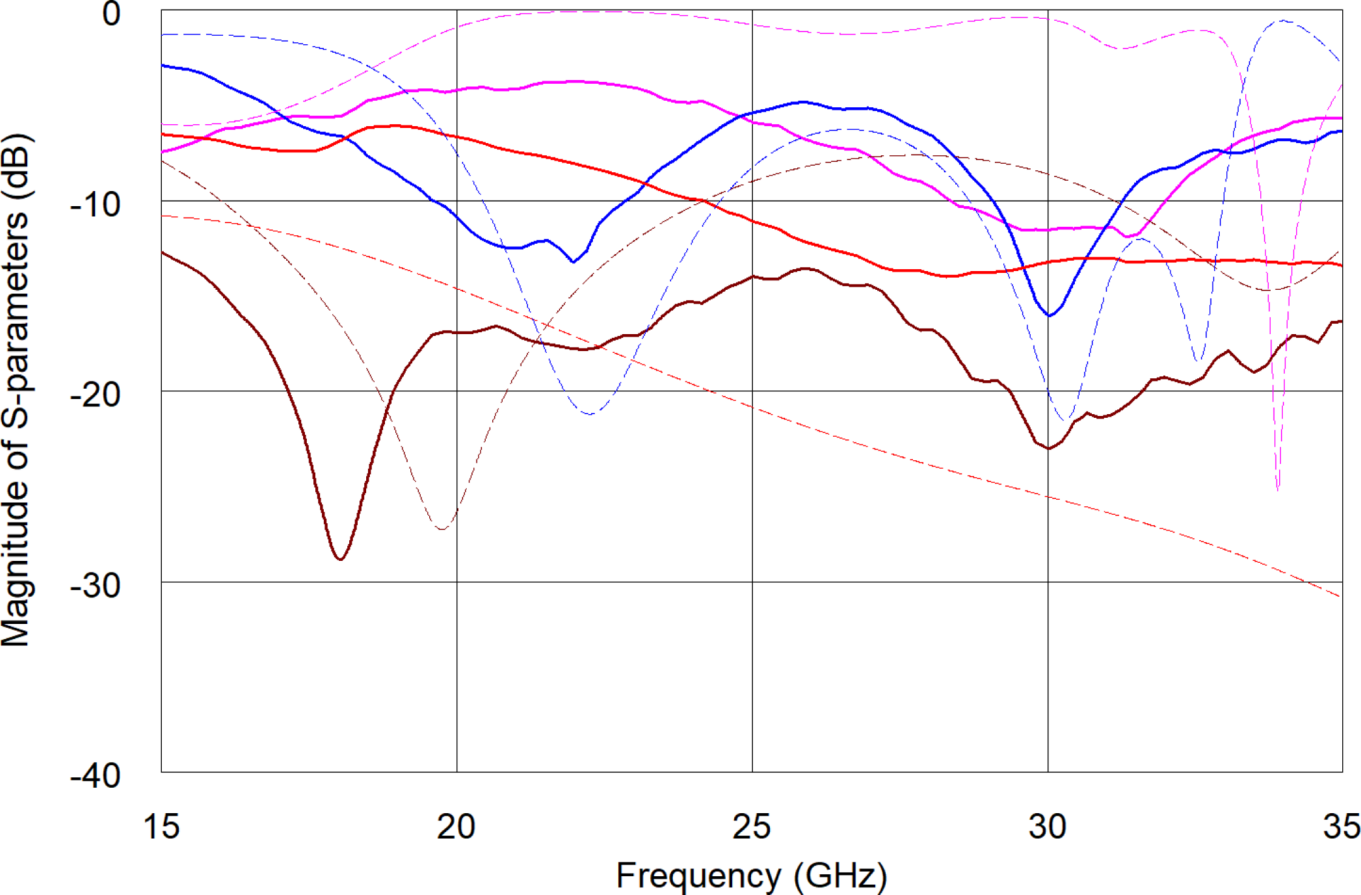
Measured magnitude of S-parameters of the sensor with no sample on top (blue and pink lines) and covered with a droplet of pure synthetic saliva (red and brown lines). Simulation results are plotted for comparison purpose with dashed lines. Blue and brown lines—reflection, pink and red lines—transmission.

The manufactured sensors set was characterized using the Agilent PNA N5224A Vector Network Analyzer connected to on-wafer probes from Cascade Microtech, Inc. The instrument was calibrated at the coax connector plane using the Short-Open-Load-Thru kit using a high-end HP/Agilent 85052D calibration kit. The measured S-parameters of an exemplary fabricated sensor unit with no cover and with 0.5 µl of pure synthetic saliva droplet are presented in Fig. 4 together with the EM simulated results (note, that EM results are obtained for water and water - glucose solution (see Sect. 2.1)). As seen, the measured reflection coefficient features similar resonances as the simulated one. The transmission level between the ports is degraded (as compared to the EM model), as the conductive layer made of sintered nanoparticles results in a structure with higher porosity and roughness than bulk silver, which translates into lower conductivity than the one assumed in the simulation. Additionally, the transmission level can be affected by the geometrical accuracy of the printed line width, which is lower in the arc area of the printed pattern.

### Detection of various glucose content in saliva

Sensitivity of the developed sensor and its application towards the detection of low concentrations of glucose in saliva was experimentally investigated by preparing and characterizing a set of mixtures with variable glucose concentrations. A range of 0 to 30 mg per 100 ml at 10 mg steps was tested to establish and assess a general trend for the data collected at points within an order of magnitude from the target application. No sample of human origin was used in this study, only synthetic materials—here saliva. All mixtures were prepared just before proceeding with the measurement campaign. At first, 1 g of glucose was dissolved in 100 ml of saliva to obtain a 1% glucose-saliva mixture. In the next step, 1% solution was further dissolved in saliva to obtain desired glucose concentrations (see Table 1). For the sake of the experimental study, 8 sensor units were chosen, 2 per glucose-saliva mixtures A-D listed in Table 1 and resulting S-parameter measurements averaged.

**Table 1:** Glucose-saliva mixture two-step preparation.

ID	Desired glucose-saliva concentration	Amount of 1% glucose-saliva mixture (g)	Amount of saliva (g)
A	0 mg/100 ml	0	100
B	10 mg/100 ml	1	99
C	20 mg/100 ml	2	98
D	30 mg/100 ml	3	97

For the glucose detection capability assessment and trend establishment, microwave measurements were carried out in a two-step process, i.e., S-parameters were collected for each sensor with no liquid cover and then after pouring 0.5 µL of the desired glucose-saliva mixtures. All measurements were carried out at a room temperature of 25 °C. A photograph of the sensor with a drop of glucose-saliva mixture on top is shown in Fig. 3b,c for two different units. As seen, the droplet spreads only partially on the glass surface covering the sensing region and is kept by the ground area placed around in a repeatable fashion as intended. The sensor features a similar construction as an interdigital capacitor, thus the greatest amount of the electric field is enclosed within the measured glucose-saliva solution covering the sensor. Thus, the transmission coefficient is directly affected by the MUT and having measured transmissions between #1 and #2 port (*S*_21_) for an empty sensor with no cover and for the sensor with appropriate glucose-saliva mixture, the transmission coefficient change for each sensor unit in a wide frequency range can be determined:

1 $$\Delta {S_{21}}\left( f \right) = {\rm{ }}\left| {{S_{21}}^{empty} - {S_{21}}^{mixture}} \right|$$

Since two sensor units were used per each concentration namely *unit_1* and *unit_2*, finally a mean value of *ΔS*_*21*_*(f)* was calculated:

2 $$\Delta {S_{21}}^{mean}\left( f \right){\rm{ }} = {\rm{ }}(\Delta {S_{21}}{\left( f \right)^{unit\_1}} + \Delta {S_{21}}{\left( f \right)^{unit\_2}})/{\rm{2}}$$

**Fig. 5:**
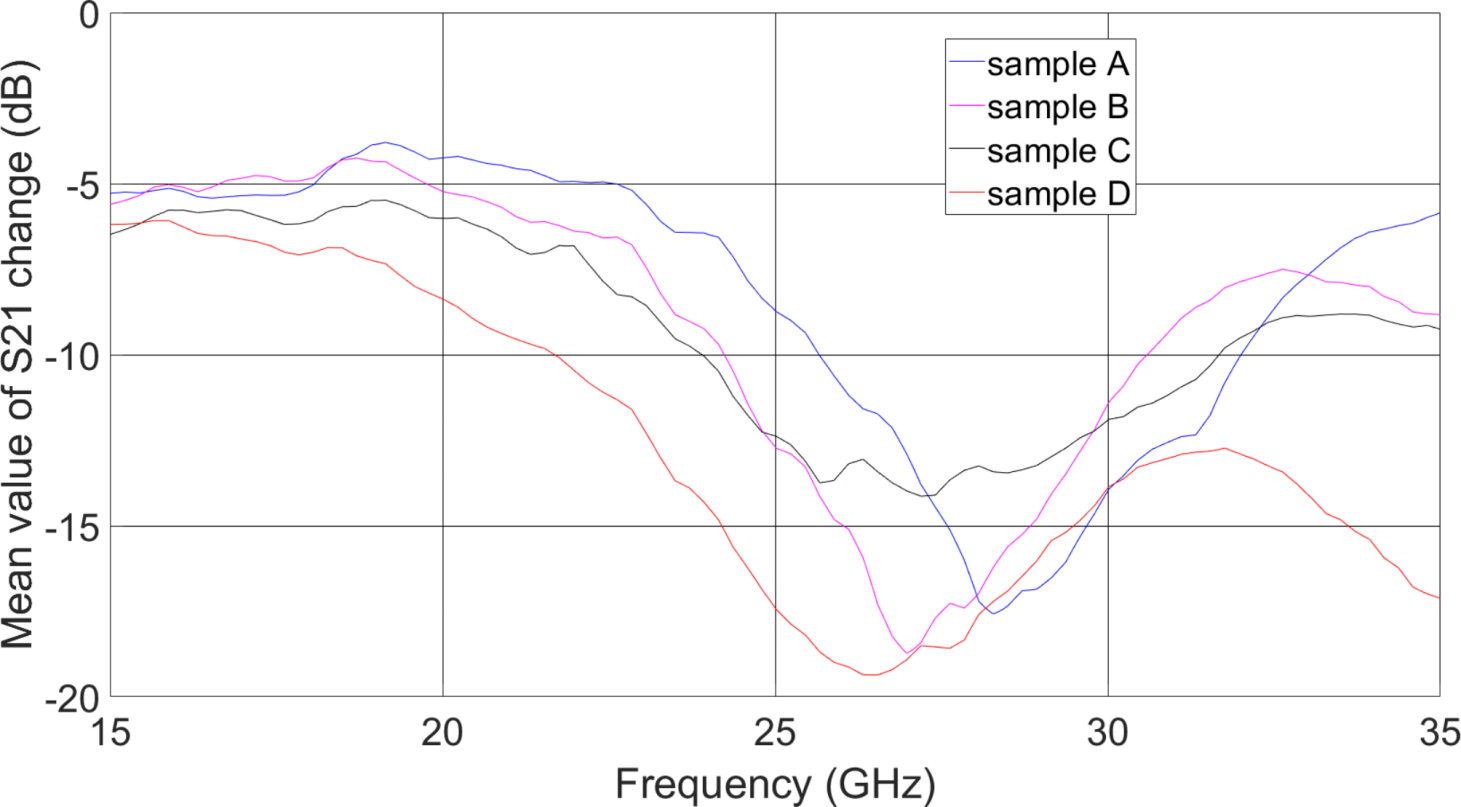
The determined based on measured S-parameters *ΔS*_*21*_^*mean*^ in a wide frequency range for concentrations A–D listed in Table 1.

The determined *ΔS*_*21*_^*mean*^*(f)* for concentrations A–D is presented in Fig. 5. As seen, all the glucose–saliva concentrations can be easily distinguished in a broad frequency range.

### Measured data analysis

**Fig. 6:**
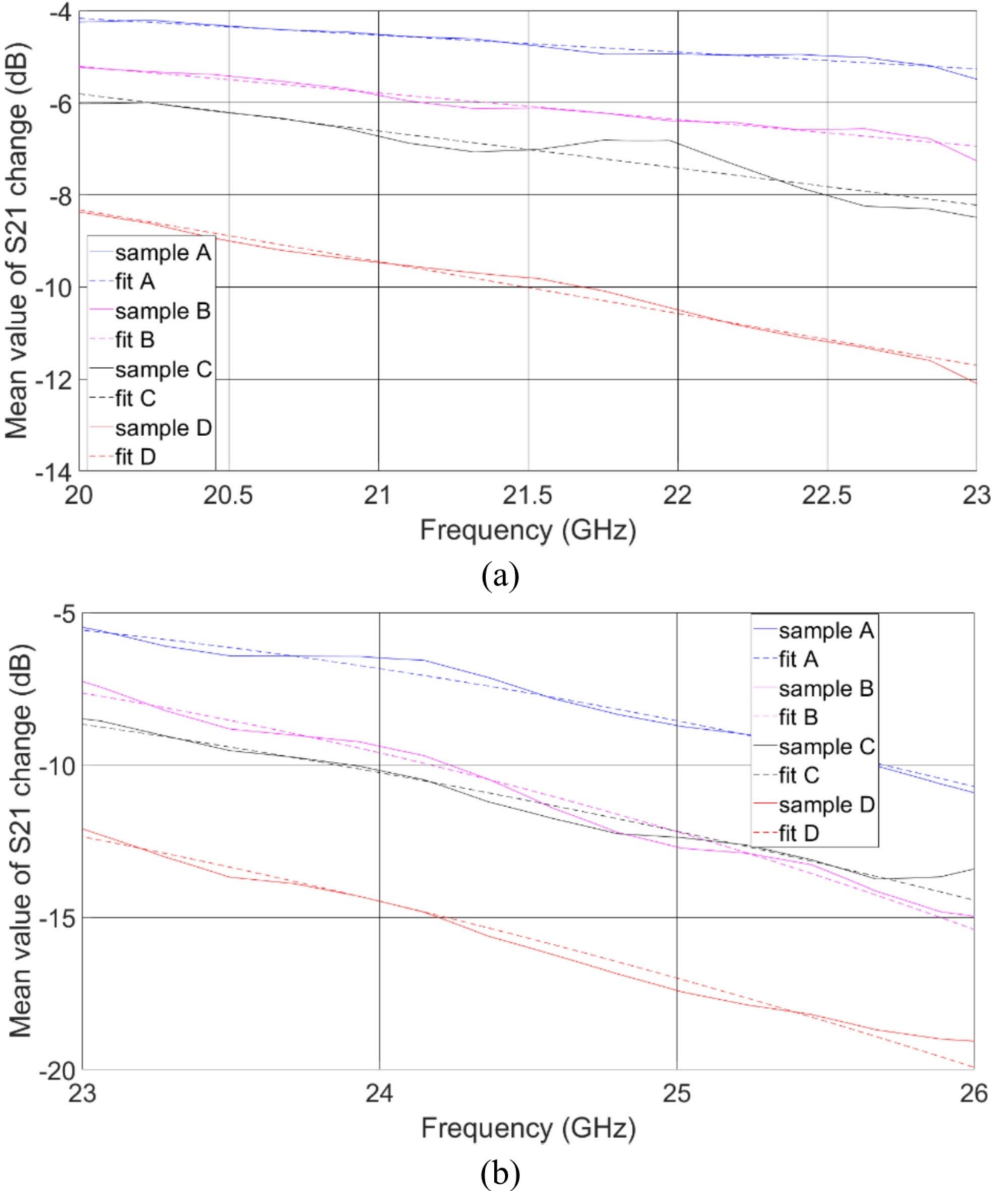
Close-up looks on *ΔS*_*21*_^*mean*^ in a wide frequency range together with the fitting linear (**a**) and quadratic (**b**) functions having coefficients as listed in Tables 2 and 3, respectively.

**Fig. 7:**
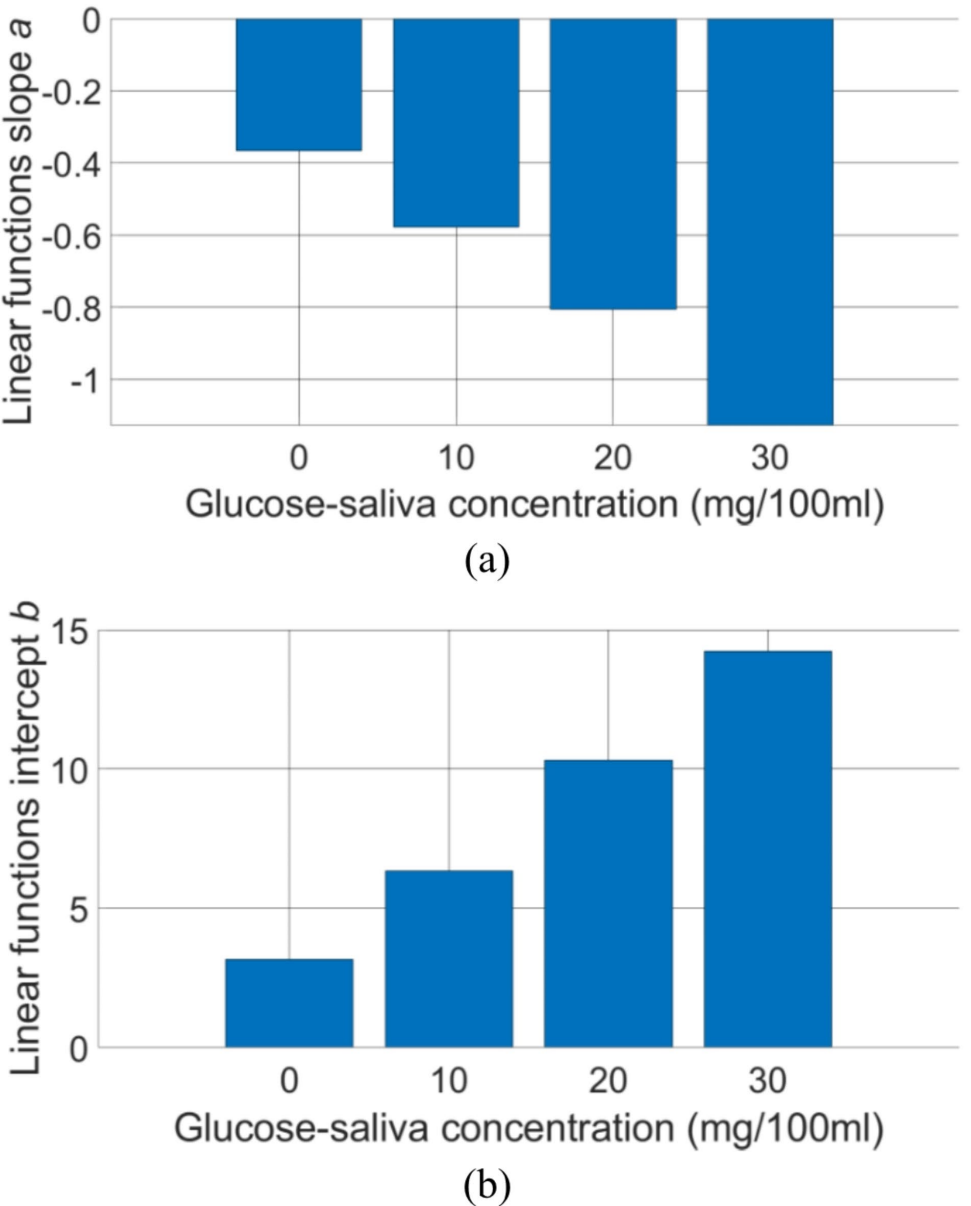
Measured transfer function for the developed sensor demonstrator where the slopes *a*_1_ and the intercept points *b*_1_ of the fitted linear functions are used as a Figure-of-Merit to quantitively sense the glucose concentration in saliva.

To relate the obtained frequency characteristics with the measured glucose concentrations in saliva, the *ΔS*_*21*_^*mean*^*(f)* characteristics were fitted in the frequency range 20–23 GHz with linear functions *a*_1_*f* + *b*_1_, whereas in the frequency range 23–26 GHz using parabolic functions *a*_2_*f*^2^ + *b*_2_*f* + *c*_2_. The results of curve fitting are presented in Fig. 6. The determined fitted linear and parabolic functions are listed in Tables 2 and 3, respectively. The determined slopes *a*_1_ and intercept points *b*_1_ of the linear fitted functions are presented in Fig. 7 and are considered as a Figure-of-Merit (FoM) in this study. As seen, the slopes’ values decrease monotonically, and the intercept points’ values increase monotonically with the increase of glucose content in saliva-glucose mixture. In the case of parabolic fitted functions, it is possible to determine the position of vertex V of the parabola, which also can be used as FoM as:

3 $$\:V=\left(x,y\right)=\left(\frac{-b}{2a},\frac{4ac-{b}^{2}}{4a}\right)$$

**Fig. 8:**
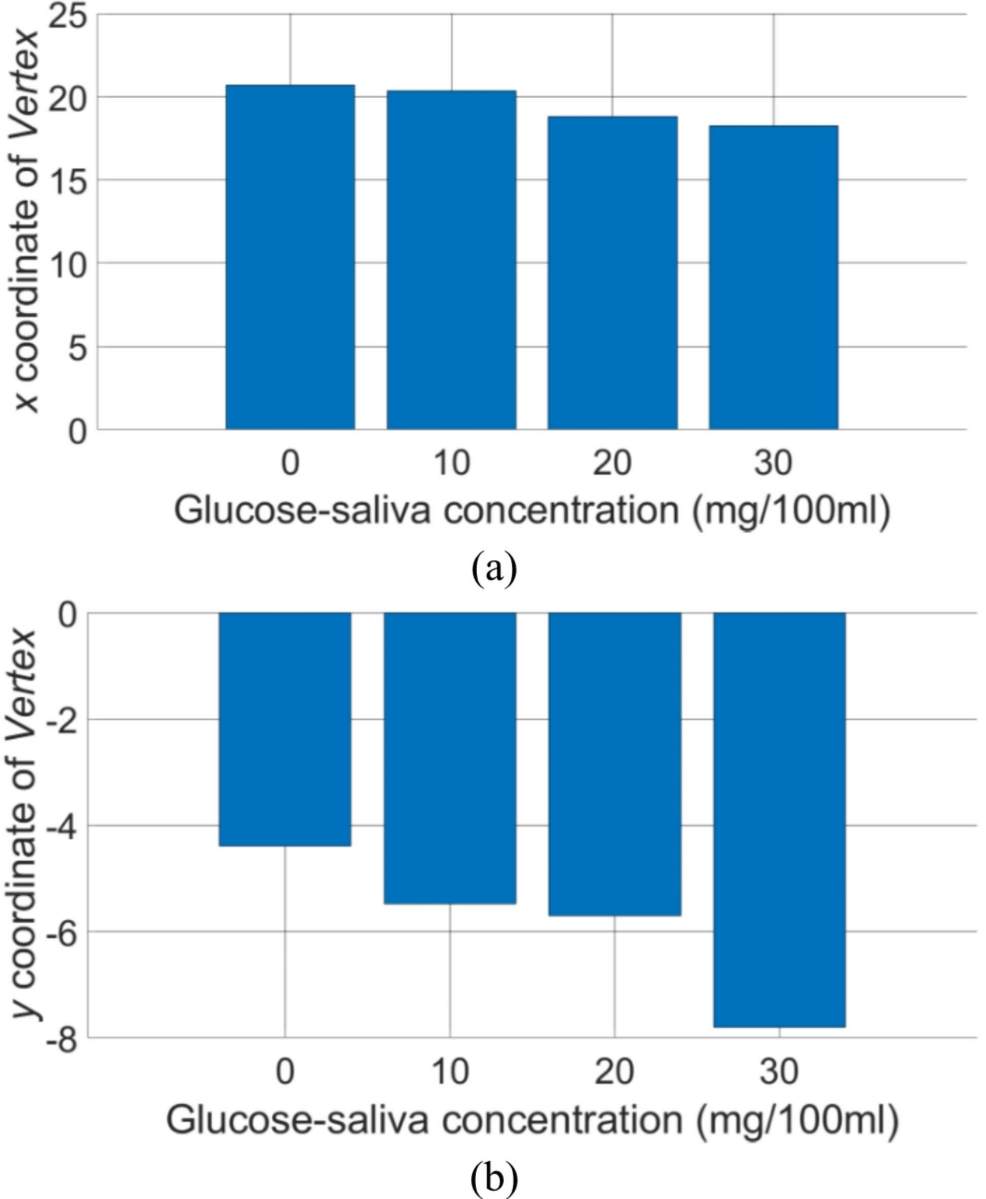
Measured transfer function for the developed sensor demonstrator where the *x* and *y* coordinates of the fitted parabola’s vertex is used as a Figure-of-Merit to quantitively sense the glucose concentration in saliva.

The calculated (*x*, *y*) coordinates of vertexes for the collected experimental data are shown in Table 3 and visualized in Fig. 8 using bar graphs. Both *x* and *y* coordinates are monotonically decreasing with the increasing glucose concentration in saliva, however higher changes are observed on the *y* coordinate.

**Table 2:** Coefficients of linear functions *a*_1_*f* + *b*_1_, fitting *ΔS*_*21*_^*mean*^*(f)* for samples A-D.

ID	a_1_	b_1_
A	− 0.37	3.17
B	− 0.58	6.34
C	− 0.81	10.31
D	− 1.13	14.23

**Table 3:** Coefficients of quadratic functions *a*_2_*f*^2^ + *b*_2_*f* + *c*_2_ fitting *ΔS*_*21*_^*mean*^*(f)* for samples A–D and determined coordinates (*x*, *y*) of vertex *V*.

ID	a_2_	b_2_	c_2_	V(x, y)	
				x	y
A	− 0.2249	9.3124	− 100.7758	20.6997	− 4.3939
B	− 0.3127	12.7353	− 135.1600	20.3659	− 5.4768
C	− 0.1690	6.3581	− 65.4955	18.8089	− 5.7013
D	− 0.2018	7.3677	− 75.0375	18.2509	− 7.8035

## Discussion

The developed microwave sensor was validated towards biomedical applications and a detectability of saliva glucose level as low as 10 mg per 100 ml was demonstrated. Determination of glucose content relies on broadband assessment of sensor`s transmission coefficient slope past the first resonance peak. The location and magnitude of the resonance is altered by the varying electrical properties of the deposited droplet. Two approaches to data analysis were presented exploiting the linear and quadratic regions of the resonance slope. In both cases a set of two parameters are obtained providing redundant information that improves the robustness of detection. It is seen from Figs. 7 and 8 that the FoM exhibits a monotonic trend with increasing concentration of glucose enabling unambiguous determination of the contend. Importantly, as seen in e.g. Fig. 6 traces for pure saliva and 10 mg per 100 ml mix are distant from each other by of 2 dB to 4 dB. It is extrapolated that for order of magnitude smaller concentration the difference should be also an order of magnitude smaller, still enabling to distinguish between pure saliva and 1 mg per 100 ml mix since typical S-params measurement uncertainty is in the range of 0.1 to 0.2 dB. For comparison purposes, the sensor shown in^11^ can detect glucose down to 0.1 mg per 100 ml, whereas the sensing circuit shown in^20^ allows for detection of glucose saliva equal to 3.6 mg per 100 ml. Even though the detection threshold obtained here is higher than the saliva glucose level threshold between diabetic and non-diabetic persons according to^16,30^, it is of the same order. It needs to be underlined, that the sensor features simple construction and low-cost realization in comparison to^11,20^, or^21^. Moreover, no surface treatment or chemical modification of the sensing element was used and such an approach can be in the future used to further increase the sensitivity of the sensor to glucose level changes. Therefore, it can be concluded that the proposed sensing approach is promising direction towards the development of modern-day diabetes management tools.

## Conclusion

A novel non-invasive glucose level detection approach through glucose concentration sensing in saliva was introduced and studied in this paper. An application-specific highly sensitive microwave biosensor was proposed based on a micrometer-scale interdigital capacitor in transmission mode implemented in a single-layer coplanar transmission line technique using 3D printed metal-on-glass technology. A K-band demonstrator was designed, fabricated, and experimentally tested. The required feature size of the metal pattern was obtained using Aerosol Jet Printing technology and an ultrasonically atomized silver-nanoparticle ink. For verification of the sensor’s abilities to detect saliva-glucose, samples of saliva with different concentrations of glucose were prepared – 0, 10, 20, and 30 mg/100 ml which are in the ballpark close to (non)-diabetic threshold. The sensor’s measured transmission under exposition to the MUT in relation to the empty sensor is used to convey the concentration information. Two fitting approaches were studied i.e., using linear and parabolic functions in different frequency regions of sensor operation to obtain redundant information, which could be used as a Figure-of-Merit in glucose amount detection in saliva. The obtained transfer functions presented a monotonically changing character with increasing concentrations of glucose in the solution. The obtained detectability threshold and resolution certified the applicability of this sensor in detecting saliva-glucose levels as well as further directions of development were identified.

## Data Availability

The authors declare that the data supporting the findings of this study are available within the paper or can be shared on-demand by reaching out to the corresponding author prof. Ilona Piekarz.

## Declarations

### Declaration of human involvement

The authors declare that the presented study does NOT involve any human participants, their data or biological material. Only artificial saliva was used as test samples.
